# Association of Hospital-Level Differences in Care With Outcomes Among Patients With Acute ST-Segment Elevation Myocardial Infarction in China

**DOI:** 10.1001/jamanetworkopen.2020.21677

**Published:** 2020-10-23

**Authors:** Haiyan Xu, Yuejin Yang, Chuangshi Wang, Jingang Yang, Wei Li, Xuan Zhang, Yunqing Ye, Qiuting Dong, Rui Fu, Hui Sun, Xinxin Yan, Xiaojin Gao, Yang Wang, Xuan Jia, Yi Sun, Yuan Wu, Jun Zhang, Wei Zhao, Marc S. Sabatine, Stephen D. Wiviott

**Affiliations:** 1 Coronary Heart Disease Center, Department of Cardiology, Fuwai Hospital, National Center for Cardiovascular Diseases, Chinese Academy of Medical Science and Peking Union Medical College, Beijing, China; 2 Medical Research and Biometrics Center, Fuwai Hospital, National Center for Cardiovascular Diseases, Chinese Academy of Medical Science and Peking Union Medical College, Beijing, China; 3 Information Technology Center, Fuwai Hospital, Beijing, China; 4 Thrombolysis in Myocardial Infarction Study Group, Cardiovascular Division, Brigham and Women’s Hospital, Harvard Medical School, Boston, Massachusetts

## Abstract

**Importance:**

The incidence of acute myocardial infarction has increased over the past decades in China, and management challenges include an unbalanced economy, disparate resources, and variable access to medical care across the nation.

**Objective:**

To examine the variations in care and outcomes of patients with ST-segment elevation myocardial infarction among 3 levels of hospitals in the typical Chinese public hospital model.

**Design, Setting, and Participants:**

This cross-sectional study used data from the China Acute Myocardial Infarction Registry to compare the differences in care and outcomes among patients at 108 hospitals from 31 provinces and municipalities throughout mainland China. Participants included patients with ST-segment elevation myocardial infarction directly admitted to hospitals between January 2013 and September 2014. Data analyses were performed from June 2015 to June 2019.

**Exposures:**

Care in province-level, prefecture-level, or county-level hospitals in China.

**Main Outcomes and Measures:**

The primary outcome was in-hospital mortality. Secondary outcomes included presentation, treatments, and major complications.

**Results:**

A total of 12 695 patients (9593 men [75.6%]; median [interquartile range] age, 63 [54-72] years) were included; 3985 were at province-level hospitals, 6731 were at prefecture-level hospitals, and 1979 were at county-level hospitals. Compared with patients admitted to province-level hospitals, those admitted to prefecture-level and county-level hospitals were older (median [interquartile range] age, 61 [52-70] years vs 63 [54-72] years and 65 [57-75] years) and more likely to be women (815 women [20.5%] vs 1620 women [24.1%] and 667 women [33.7%]). Patients in prefecture-level and county-level hospitals were less likely to use ambulances compared with patients at province-level hospitals (11.6% [95% CI, 10.8%-12.4%] and 12.0% [95% CI, 10.6%-13.5%] vs 19.4% [95% CI, 18.1%-20.7%]; *P* < .001) and were less likely to experience early presentation, with onset-to-arrival times less than 12 hours for 75.3% (95% CI, 73.9%-76.6%) of patients at province-level hospitals, 70.8% (95% CI, 69.7%-71.9%) of patients at prefecture-level hospitals, and 69.8% (95% CI, 67.7%-71.8%) of patients at county-level hospitals (*P* < .001). The rates of reperfusion therapy were significantly lower in low-level hospitals (54.3% [95% CI, 53.1%-55.5%] for prefecture-level hospitals and 45.8% [95% CI, 43.6%-48.1%] for county-level hospitals) compared with province-level hospitals (69.4% [95% CI, 67.9%-70.8%]; *P* < .001). There was a progressively higher rate of in-hospital mortality at the 3 levels of hospitals: 3.1% (95% CI, 2.6%-3.7%) for province-level hospitals, 5.3% (95% CI, 4.8%-5.9%) for prefecture-level hospitals, and 10.2% (95% CI, 8.9%-11.7%) for county-level hospitals (*P* for trend < .001). After adjustment for patient characteristics, presentation, hospital facility, and treatments, the odds of death remained higher in prefecture-level (odds ratio, 1.39 [95% CI, 1.06-1.84]) and county-level (odds ratio, 1.43 [95% CI, 0.97-2.11]) hospitals compared with province-level hospitals (*P* for trend = .04).

**Conclusions and Relevance:**

These findings suggest that there are significant variations in care and outcomes of patients among the 3 levels of hospitals in China. More efforts should be made to address the identified gaps, particularly in the prefecture-level and county-level hospitals. This work can inform national quality improvements efforts in China and in other developing countries.

## Key Points

**Question:**

What are the differences in care and outcomes of patients with ST-segment elevation myocardial infarction among 3 vertical levels of hospitals in China?

**Findings:**

In this cross-sectional study using data from the China Acute Myocardial Infarction Registry, which included 108 hospitals at the province, prefecture, and county levels, compared with patients in province-level hospitals, the rates of reperfusion therapy were lower among those in prefecture-level and county-level hospitals (69.4% vs 54.3% vs 45.8%). In-hospital mortality rates progressively increased among the 3 levels of hospitals, from 3.1% at the province level to 5.3% at the prefecture level to 10.2% at the county level.

**Meaning:**

These findings suggest that more efforts should be made to address the gaps in care and outcomes of ST-segment elevation myocardial infarction for national quality improvement in China.

## Teaser

This cross-sectional study examines the variations in care and outcomes of patients with acute ST-segment elevation myocardial infarction (STEMI) at 3 different levels of hospitals—province, prefecture, and county—in China.

## Introduction

Ischemic heart disease is the leading cause of death in both developed and developing countries like China, largely due to acute coronary syndromes, particularly acute myocardial infarction (AMI).^1^ The mortality rate attributed to AMI remains variable across different regions worldwide.^2,3,4^ A decrease in deaths due to AMI has been seen in some countries, mainly because of the improvements in emergency medical services (EMSs), widespread adoption of early reperfusion therapy, and optimal medication usage in routine practice.^5,6,7,8,9,10^ On the other hand, the mortality rate associated with AMI is steady or even elevated in developing countries, which account for more than 80% of global deaths due to ischemic heart disease.^2,3^ The data on deaths in China demonstrated an upward trend by 5.6-fold in AMI-related deaths in both urban and rural populations from 1987 to 2014, with acceleration from 2006 onward and a more rapid increase in rural regions.^11^ A retrospective study^12^ also indicated that the in-hospital mortality rate for ST-segment elevation myocardial infarction (STEMI) in China had not changed within 10 years since 2001 despite rapid progresses in reperfusion therapy. China still faces challenges in providing optimal and equitable management strategies for all patients across the nation because of the broad geography and unbalanced economic development. In addition, disparate access to medical care may also lead to variations in AMI care provision, treatment patterns, and outcomes.

The major public medical system in China follows a traditional structure based on vertically administrative models of province, prefecture, and county in the order of decreasing size and level. The China AMI (CAMI) registry is a prospective, nationwide, multicenter, observational study for AMI care in these 3 levels of hospitals. The present cross-sectional study investigates the variations in care and outcomes of patients with STEMI among the 3 levels of hospitals in China.

## Methods

### Overview of CAMI Registry and Study Population

This study was approved by the institutional review board central committee at Fuwai Hospital. Written informed consent was obtained from eligible patients. This report follows the Strengthening the Reporting of Observational Studies in Epidemiology (STROBE) reporting guideline for cross-sectional studies.

The design of the CAMI registry was described previously.^13^ Briefly, 108 hospitals from 31 provinces and municipalities throughout mainland China have participated in the registry since January 2013 (eAppendix 1 in the Supplement). These hospitals included 31 province-level hospitals, 45 prefecture-level hospitals in their own provinces or municipalities, and 32 county-level hospitals in these selected prefectures, with broad coverage of geographical regions, including urban and rural areas. These hospitals are the largest or central hospitals in their administrative areas. Province-level hospitals are all university-affiliated academic hospitals located in capital city of each province, prefecture-level hospitals are in medium-sized cities, and county-level hospitals are in the smallest cities, usually with surrounding rural areas. Staffing ratios of cardiologists and cardiothoracic surgeons are in decreasing order from province to prefecture to county levels. In province-level, prefecture-level, and county-level hospitals, the median bed numbers in cardiology units are 122, 83, and 47, respectively; 100%, 96%, and 78% of hospitals, respectively, have a cardiac-coronary care unit (CCU); and 100%, 93%, and 44% of hospitals, respectively, have a catheterization laboratory.^13^ The vertical administrative relationship of the 3 levels of hospitals reflects the hierarchical performance of the current medical care system (eFigure 1 in the Supplement), thus making hospital-level comparisons appropriate.

Patients with a primary diagnosis of AMI including STEMI and non-STEMI admitted to participating hospitals within 7 days after the onset of ischemic symptoms were consecutively enrolled into the registry. The final diagnosis had to meet the third Universal Definition for Myocardial Infarction, including types 1, 2, 3, 4b, and 4c. Type 4a and type 5 AMIs were not eligible for the CAMI registry.^14^

### Data Collection and Management

Comprehensive collection of data, including patient demographic factors, risk factors, medical history, prehospital medical contact, presentation, status at admission, vital signs, reperfusion therapy and reasons for it, medications, procedures, and events, was conducted. All information was collected using a standardized set of variables and predefined, standard, unified definitions, systematic data entry and transmission procedures, and rigorous data quality control. Data were collected, validated, and submitted through a secure, web-based electronic data capture system. Enrollment, data collection, and follow-up were all performed by trained physicians at each participating site in a real-time manner, to ensure data accuracy and reliability. Senior cardiologists were responsible for the data quality control. Periodic database checking was undertaken. Hospital sites underwent random on-site audits for the accuracy of diagnosis and variables based on medical records.

### Variables in Care and Outcomes

The key variable in care for patients with STEMI was reperfusion therapy, including primary percutaneous coronary intervention (PCI) or fibrinolysis. We analyzed the percentage of patients undergoing reperfusion therapy among all patients and among the eligible patients who presented within 12 hours after symptom onset. Coronary angiography, stent implantation, elective PCI, coronary artery bypass grafting, intra-aortic balloon pump use, and medications, including traditional Chinese medicines, used during hospitalization were also assessed. The primary outcome was in-hospital mortality. Other outcomes included the complication rates of heart failure, cardiogenic shock, mechanical complications, severe arrhythmias, reinfarction, cerebrovascular accident or stroke, and nonintracranial hemorrhage bleedings (detailed definitions are shown in eAppendix 2 in the Supplement).

### Statistical Analysis

Patient characteristics, medical contact, treatments, and the rates of in-hospital outcomes were compared among the 3 levels of hospitals. Continuous variables were expressed by mean (SD) or median (interquartile range) and were compared by analysis of variance or the Kruskal-Wallis H test as appropriate. Categorical variables were expressed as percentages with 95% CIs, and comparison was performed with the χ^2^ test. We also performed the Cochran-Armitage trend test for the rates of in-hospital outcomes among the 3 levels of hospitals. Multivariable logistic regression models were used to examine the differences in the odds of in-hospital outcomes among the 3 levels of hospitals by adjusting for confounding variables. Models 1 to 6 were fitted by adjusting for patient characteristics, medical contact, clinical status at admission, hospital facilities, reperfusion therapy, medications, and intra-aortic balloon pump use during hospitalization, as shown in eAppendix 2 in the Supplement. Odds ratios (ORs) and 95% CIs were calculated. In addition, linear trend tests were conducted for the ORs of death, heart failure, and cardiogenic shock among the 3 levels of hospitals. In-hospital mortality among the 3 levels of hospitals was also compared across 2 subgroups by onset-to-admission time of 12 hours or less vs longer than 12 hours and by receipt of reperfusion therapy or not. Multivariable logistic regression models were also used for the assessment of associated factors with in-hospital mortality. All the variables were missing in fewer than 10% of all cases, and multivariable outcomes analyses were based on the complete data. We also used multiple imputations for baseline variables to perform logistic regression to assess the outcomes as sensitivity analysis. A 2-tailed *P* < .05 was considered significant. Statistical analyses were performed using SAS statistical software (version 9.4 for Windows, SAS Institute) from June 2015 to June 2019.

## Results

### Patients in the Analysis

**Figure 1.**
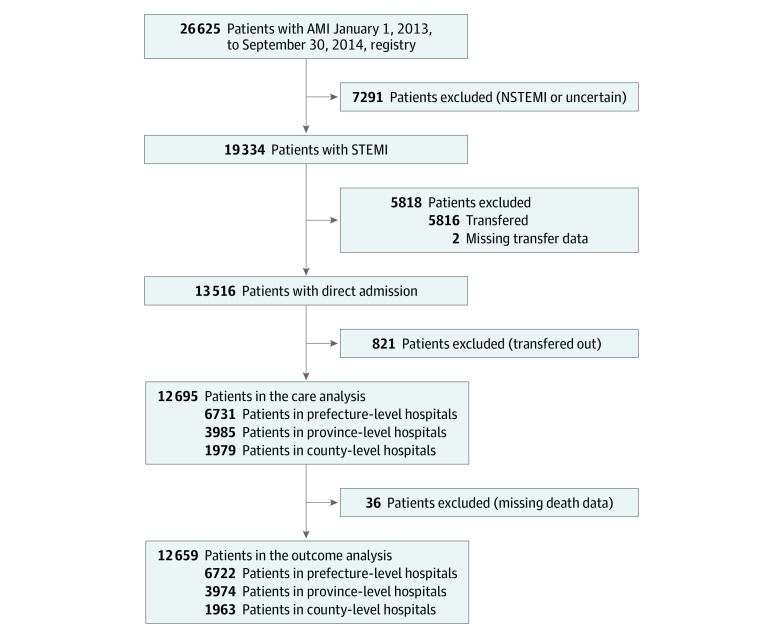
Study Cohort Flow Diagram AMI indicates acute myocardial infarction; NSTEMI, non–ST-segment elevation myocardial infarction; and STEMI, ST-segment elevation myocardial infarction.

Of 19 334 patients with acute STEMI registered in the CAMI registry from January 1, 2013, through September 30, 2014, we excluded 5816 patients who transferred in, 821 patients who transferred out, and 2 patients with missing transfer data, to obtain the real prehospital information and to eliminate referral bias in comparison of care and outcomes among the 3 levels of hospitals. Thus, 12 695 patients (9593 men [75.6%]; median [interquartile range] age, 63 [54-72] years) with direct admission formed the core cohort for the care analysis, with 3985 patients in province-level hospitals, 6731 patients in prefecture-level hospitals, and 1979 patients in county-level hospitals (Figure 1). Moreover, 12 659 patients were included in the in-hospital outcomes analysis after further exclusion of 36 patients because of missing data on death.

### Baseline Characteristics, Medical Contact, and Presentation

Compared with patients admitted to province-level hospitals, the patients admitted to prefecture-level and county-level hospitals were older (median [interquartile range] age, 61 [52-70] years vs 63 [54-72] years and 65 [57-75] years) and more likely to be women (815 women [20.5%] vs 1620 women [24.1%] and 667 women [33.7%]), but less likely to have obesity, diabetes, dyslipidemia, previous PCI, and peripheral arterial disease and to be current smokers (Table 1). Compared with patients in province-level hospitals, those in prefecture-level and county-level hospitals were significantly less likely to arrive at the hospital within 12 hours from symptom onset (75.3% [95% CI, 73.9%-76.6%] vs 70.8% [95% CI, 69.7%-71.9%] vs 69.8% [95% CI, 67.7%-71.8%]; *P* < .001), and they had lower rates of ambulance transportation use (19.4% [95% CI, 18.1%-20.7%] vs 11.6% [95% CI, 10.8%-12.4%] vs 12.0% [95% CI, 10.6%-13.5%]; *P* < .001). Overall, 72.0% of patients (9147 of 12 695 patients) arrived at the hospital within 12 hours after symptom onset, and 14.1% of patients (141 of 12 440 patients) used ambulances. More patients in county-level hospitals presented with heart failure, cardiogenic shock, cardiac arrest, and Killip class III or IV heart failure (Table 1 and eTable 1 and eTable 2 in the Supplement).

**Table 1.** Baseline Characteristics of Patients With ST-Segment Elevation Myocardial Infarction Among the 3 Levels of Hospitals

Characteristics	Patients, No./total No. (%)				*P* value^a^
	Total (N = 12 695)	Province level (n = 3985)	Prefecture level (n = 6731)	County level (n = 1979)	
Age, median (IQR), y	63 (54-72)	61 (52-70)	63 (54-72)	65 (57-75)	<.001
≥75 y	2270/12 510 (18.1)	591/3945 (15.0)	1219/6619 (18.4)	460/1946 (23.6)	<.001
Male	9593/12 695 (75.6)	3170/3985 (79.5)	5111/6731 (75.9)	1312/1979 (66.3)	<.001
Risk factors and medical history					
Body mass index, mean (SD)^b^	24.5 (10.7)	25.1 (15.6)	24.3 (8.4)	23.7 (3.4)	<.001
Body mass index ≥25	4282/12 049 (35.5)	1516/3763 (40.3)	2154/6388 (33.7)	612/1898 (32.2)	<.001
Current smoker	5601/12 401 (45.2)	1968/3849 (51.1)	2856/6606 (43.2)	777/1946 (39.9)	<.001
Hypertension	6040/12 225 (49.4)	1926/3781 (50.9)	3187/6547 (48.7)	927/1897 (48.9)	.08
Diabetes history	2267/11 958 (19.0)	789/3687 (21.4)	1200/6446 (18.6)	278/1825 (15.2)	<.001
Known dyslipidemia	789/10 792 (7.3)	335/3291 (10.2)	356/6011 (5.9)	98/1490 (6.6)	<.001
Prior myocardial infarction	792/11 673 (6.8)	234/3397 (6.9)	446/6406 (7.0)	112/1870 (6.0)	.31
Prior percutaneous coronary intervention	558/12 074 (4.6)	220/3579 (6.1)	287/6581 (4.4)	51/1914 (2.7)	<.001
Prior heart failure	193/11 845 (1.6)	54/3435 (1.6)	97/6518 (1.5)	42/1892 (2.2)	.10
Prior stroke	1094/11 970 (9.1)	299/3484 (8.6)	610/6555 (9.3)	185/1931 (9.6)	.37
Peripheral artery disease	54/11 898 (0.5)	22/3460 (0.6)	30/6525 (0.5)	2/1913 (0.1)	.01
Presentation					
Means of transport					
Self or family	10 551/12 440 (84.8)	3081/3856 (79.9)	5768/6616 (87.2)	1702/1968 (86.5)	<.001
Ambulance	1748/12 440 (14.1)	747/3856 (19.4)	765/6616 (11.6)	236/1968 (12.0)	
In-hospital	141/12 440 (1.1)	28/3856 (0.7)	83/6616 (1.3)	30/1968 (1.5)	
Onset-to-arrival time					
<3 h	3903/12 695 (30.7)	1153/3985 (28.9)	2092/6731 (31.1)	658/1979 (33.2)	<.001
3-12 h	5244/12 695 (41.3)	1847/3985 (46.3)	2674/6731 (39.7)	723/1979 (36.5)	
13-24 h	1098/12 695 (8.6)	323/3985 (8.1)	584/6731 (8.7)	191/1979 (9.7)	
1-7 d	2343/12 695 (18.5)	634/3985 (15.9)	1322/6731 (19.6)	387/1979 (19.6)	
Uncertain	107/12 695 (0.8)	28/3985 (0.7)	59/6731 (0.9)	20/1979 (1.0)	
Anterior myocardial infarction	6511/12 467 (52.2)	2014/3876 (52.0)	3437/6620 (51.9)	1060/1971 (53.8)	.32
Heart failure at admission	1695/12 347 (13.7)	468/3824 (12.2)	878/6574 (13.4)	349/1949 (17.9)	<.001
Cardiogenic shock at admission	482/12 396 (3.9)	107/3852 (2.8)	246/6587 (3.7)	129/1957 (6.6)	<.001
Cardiac arrest	175/12 429 (1.4)	50/3861 (1.3)	83/6602 (1.3)	42/1966 (2.1)	.02
Systolic blood pressure, median (IQR), mm Hg	128 (110-144)	128 (111-143)	127 (110-144)	130 (110-147)	.39
Heart rate, median (IQR), beats/min	76 (65-87)	76 (66-86)	76 (65-87)	76 (65-90)	.005
Killip class III or IV heart failure	955/12 419 (7.7)	231/3856 (6.0)	538/6596 (8.2)	186/1967 (9.4)	<.001

Abbreviation: IQR, interquartile range.

^a^ *P* values indicate between-hospital comparisons of baseline characteristics for patients with ST-segment elevation myocardial infarction among 3 levels of hospitals.

^b^ Body mass index is calculated as weight in kilograms divided by height in meters squared.

### Reperfusion Therapy, Procedures, and Medications

Rates of reperfusion therapy in province-level hospitals were significantly higher than those in prefecture-level and county-level hospitals among all patients (69.4% [95% CI, 67.9%-70.8%] vs 54.3% [95% CI, 53.1%-55.5%] vs 45.8% [95% CI, 43.6%-48.1%]; *P* < .001; the rate of perfusion therapy for all patients was 57.5% [7123 of 12 363 patients]) and among the eligible patients admitted within 12 hours after symptom onset (88.6% [95% CI, 87.3%-89.8%] vs 80.1% [95% CI, 78.8%-81.3%] vs 72.6% [95% CI, 70.0%-75.2%]; *P* < .001). Rates of primary PCI at province-level hospitals were significantly higher than rates at prefecture-level and county-level hospitals among all patients (65.7% [95% CI, 64.2%-67.2%] vs 42.2% [95% CI, 41.0%-43.4%] vs 20.2% [95% CI, 18.4%-22.1%]; *P* < .001) and among eligible patients admitted within 12 hours after symptom onset (83.8% [95% CI, 82.4%-85.2%] vs 61.5% [95% CI, 60.0%-63.0%] vs 31.6% [95% CI, 29.0%-34.4%]; *P* < .001) (Table 2 and eTable 3 and eTable 4 in the Supplement). The reasons for not receiving reperfusion therapy included patients or family members refusing because of concerns about reperfusion-related complications, patient’s finances, physician’s decision, and unclear diagnosis (predominantly in lower-level hospitals) (eFigure 2 in the Supplement). Of note, time delays from admission to care were seen in the 3 levels of hospitals; 15.4% (95% CI, 6.9%-28.1%) of patients at province-level hospitals, 31.0% (95% CI, 26.6%-35.6%) of patients at prefecture-level hospitals, and 36.5% (95% CI, 30.6%-42.9%) of patients at county-level hospitals met the door-to-needle time goal of 30 minutes or less, and 32.7% (95% CI, 28.9%-36.6%) of patients at province-level hospitals, 41.5% (95% CI, 38.6%-44.5%) of patients at prefecture-level hospitals, and 29.7% (95% CI, 22.5%-37.8%) of patients at county-level hospitals met the door-to-balloon time goal of 90 minutes or less (eFigure 3 in the Supplement).

**Table 2.** Treatments for Patients With ST-Segment Elevation Myocardial Infarction in China and Among the 3 Levels of Hospitals

Treatments	Patients, No./total No. (%)				*P* value^a^
	Total (N = 12 695)	Province level (n = 3985)	Prefecture level (n = 6731)	County level (n = 1979)	
Reperfusion therapy					
Among all the patients	7123/12 363 (57.5)	2653/3824 (69.4)	3576/6589 (54.3)	894/1950 (45.8)	<.001
Primary PCI	5691/12 363 (46.0)	2514/3824 (65.7)	2783/6589 (42.2)	394/1950 (20.2)	
Fibrinolysis	1419/12 363 (11.5)	137/3824 (3.6)	782/6589 (11.9)	500/1950 (26)	
Among the eligible patients admitted within 12 h from onset	6533/7982 (81.8)	2379/2685 (88.6)	3302/4124 (80.1)	852/1173 (72.6)	<.001
Primary PCI	5159/7982 (64.6)	2251/2685 (83.8)	2537/4124 (61.5)	371/1173 (31.6)	
Fibrinolysis	1363/7982 (17.1)	126/2685 (5.0)	756/4124 (18.3)	481/1173 (41.0)	
Door-to-balloon time, median (IQR), min	113 (77-204)	123 (80-255)	109 (74-175)	124 (85-277)	.17
Door-to needle time, median (IQR), min	52 (29-100)	91 (45-187)	54 (28-94)	45 (30-100)	.15
Procedure					
Coronary angiography	7722/12 695 (60.8)	2995/3985 (75.2)	4091/6731 (60.8)	636/1979 (32.1)	<.001
Stent implantation in primary PCI	4799/5594 (85.8)	2171/2492 (87.1)	2283/2711 (84.2)	345/391 (88.2)	.004
Drug-eluting stent	4287/4755 (90.1)	1838/2155 (85.2)	2116/2260 (93.6)	333/340 (97.9)	<.001
Elective PCI	2853/12 289 (23.2)	753/3772 (20.0)	1800/6575 (27.4)	300/1942 (15.4)	<.001
Coronary artery bypass graft	53/12 456 (0.4)	30/3842 (0.8)	20/6642 (0.3)	3/1972 (0.2)	<.001
Intra-aortic balloon pump use	395/12 276 (3.2)	164/3806 (4.3)	221/6538 (3.4)	10/1932 (0.5)	<.001
Medication during hospitalization					
Aspirin	12 056/12 406 (97.2)	3758/3852 (97.6)	6425/6607 (97.2)	1873/1947 (96.2)	.02
P_2_Y_12_-receptor inhibitor	11 977/12 318 (97.2)	3754/3821 (98.2)	6411/6557 (97.8)	1812/1940 (93.4)	<.001
Statin	11 311/11 609 (97.4)	3539/3603 (98.2)	6047/6179 (97.9)	1725/1827 (94.4)	<.001
β-blocker	8623/12 283 (70.2)	2695/3806 (70.8)	4623/6541 (70.7)	1305/1936 (67.4)	.01
Angiotensin-converting enzyme inhibitor or angiotensin receptor blocker	7265/12 269 (59.2)	2267/3795 (59.7)	3807/6537 (58.2)	1191/1937 (61.5)	.03
Heparin or fondaparinux	11 091/12 137 (91.4)	3310/3758 (88.1)	6016/6459 (93.1)	1765/1920 (91.9)	<.001
Glucoprotein IIb or IIIa inhibitor	4175/11 963 (34.9)	1773/3731 (47.5)	2018/6326 (31.9)	384/1906 (20.1)	<.001
Traditional Chinese medicine	1994/12 231 (16.3)	520/3785 (13.7)	1138/6510 (17.5)	336/1936 (17.4)	<.001
Length of stay, median (IQR), d	10 (7-13)	8 (6-11)	11 (7-14)	10 (7-14)	.44

Abbreviations: IQR, interquartile range; PCI, percutaneous coronary intervention.

^a^ *P* values indicate between-hospital comparisons of treatments for patients with STEMI among 3 levels of hospitals.

Intra-aortic balloon pump was more frequently used in province-level and prefecture-level hospitals than in county-level hospitals (4.3% [95% CI, 3.7%-5.0%] vs 3.4% [95% CI, 3.0%-3.8%] vs 0.5% [95% CI, 0.2%-0.9%]; *P* < .001), despite higher rates of cardiogenic shock at presentation in county-level hospitals. We noted minor differences in medication use among 3 levels of hospitals. The usage rates for aspirin, P_2_Y_12_- receptor inhibitor, and statins during hospitalization were all high (>90%) (Table 2).

### In-Hospital Outcomes

**Figure 2.**
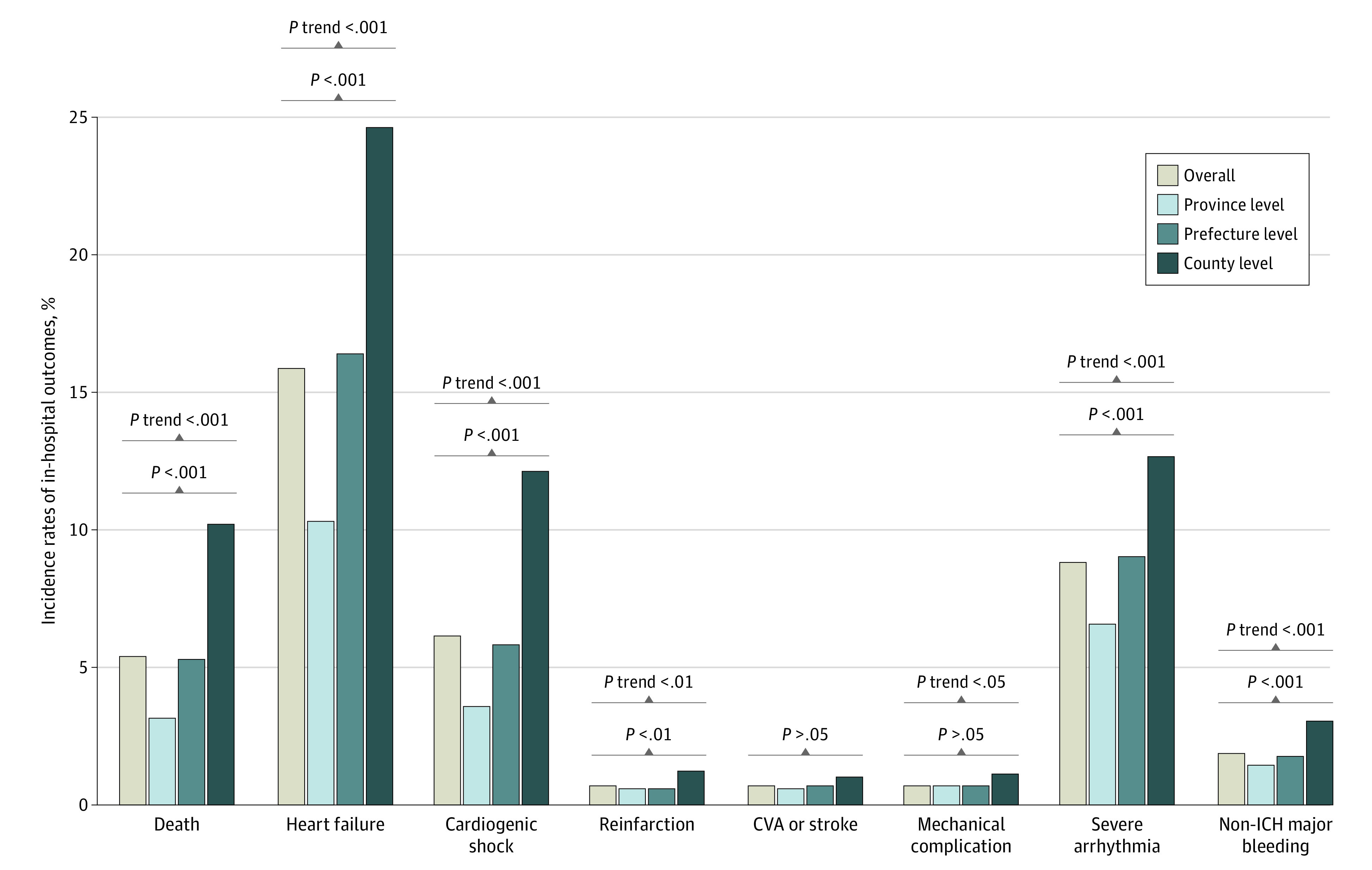
Incidence of In-Hospital Outcomes in Patients With ST-Segment Elevation Myocardial Infarction at the 3 Levels of Hospitals in China CVA indicates cerebrovascular accident; and ICH, intracranial hemorrhage.

**Figure 3.**
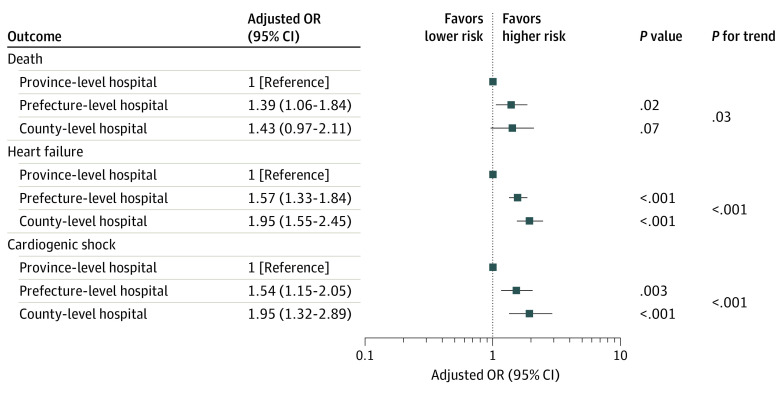
Risk-Adjusted In-Hospital Outcomes Among Patients With ST-Segment Elevation Myocardial Infarction at the 3 Levels of Hospitals in China Adjustment variables include age, sex, hypertension, diabetes, prior myocardial infarction, prior heart failure, onset-to-arrival time, means of transport, anterior-wall infarction, systolic blood pressure at admission, heart rate at admission, cardiogenic shock at admission, heart failure at admission, cardiac arrest before or at admission, Killip class of heart failure, coronary care unit, coronary catheter laboratory availability, reperfusion therapy, aspirin, P_2_Y_12_- receptor inhibitor, statin, β-blocker, angiotensin-converting enzyme inhibitor or angiotensin receptor blocker, and intra-aortic balloon pump use during hospitalization. OR indicates odds ratio.

There was a significant and progressive trend for higher in-hospital mortality among 3 levels of hospitals: 3.1% (95% CI, 2.6%-3.7%) in province-level hospitals, 5.3% (95% CI, 4.8%-5.9%) in prefecture-level hospitals, and 10.2% (95% CI, 8.9%-11.7%) in county-level hospitals (*P* for trend < .001) (Figure 2). The disparity persisted in the subsets of reperfusion therapy and onset-to-arrival time less than 12 h or not (eTable 5 and eTable 6 in the Supplement). After adjustment for patient characteristics and presentation, the odds of death was still significantly higher in prefecture-level (adjusted OR, 1.47; 95% CI, 1.14-1.89) and county-level (adjusted OR, 2.48; 95% CI, 1.85-3.31) hospitals compared with province-level hospitals (*P* < .001). After further adjustment for hospital facility and treatments, the odds of death in the 2 lower levels of hospitals were attenuated but still higher than the odds in province-level hospitals (OR, 1.39 [95% CI, 1.06-1.84] for prefecture-level hospitals and 1.43 [95% CI, 0.97-2.11] for county-level hospitals; *P* for trend = .04). Similar results were obtained in sensitivity analysis of multiple imputation (adjusted OR, 1.55 [95% CI, 1.23-1.95] for prefecture-level hospitals and 1.87 [95% CI, 1.36-2.56] for county-level hospitals; *P* < .001) (Figure 3 and eTable 7 and eTable 8 in the Supplement). Receiving treatment at the 2 lower levels of hospitals was associated with in-hospital death, but the availability of a CCU and catheterization laboratory in the hospital were protective factors against in-hospital death (eTable 9 and eTable 10 in the Supplement). Similar variations were also seen in the odds of heart failure and cardiogenic shock (Figure 3 and eTable 7 and eTable 8 in the Supplement).

## Discussion

Gaps and variations in care and outcomes for patients with STEMI exist within and across countries in the world, particularly between developed and developing countries.^1,3,15,16,17,18,19,20^ The broad geography, unbalanced economy, and varying medical service capabilities across China make the disparities even greater. The present study has several important findings. First, the prehospital delay of patients with STEMI was common and prominent, with a low ambulance use rate of only 14.1% overall. Second, the rates of reperfusion therapy, especially primary PCI, were much lower in the prefecture-level and county-level hospitals compared with the province-level hospitals. The in-hospital delay of reperfusion therapy was also obvious, with only approximately one-third reaching the time goals. Third, there existed great disparity in the in-hospital mortality rates among the 3 levels of hospitals, with a rate of 10.2% in county-level hospitals, which is 3-fold higher than the 3.1% rate in province-level hospitals. Hospital facility and reperfusion therapy partly explained hospital-level variation in mortality. To our knowledge, this is the first national report on the hospital-level differences in medical care and outcomes for patients with STEMI in China, and it reveals the gaps and challenges that China is facing. These findings establish the fundamental current status in care and outcomes of STEMI and serve as a basis to guide efforts on quality improvement in STEMI care and allocation of resources.

We selected a diverse group of 3 levels of major public hospitals in all provinces and municipalities throughout mainland China, thus making the study representative of status in the ways to seek medical care, the performance of care with the facilities for reperfusion therapy, and the outcomes of AMI in China. The facilities for AMI care are unbalanced across the 3 levels of hospitals. This prospective national registry provided a unique opportunity to evaluate the differences in prehospital contact, treatment strategies and causes, and outcomes of patients among the 3 levels of hospitals.

The CAMI registry enrolled patients with AMI admitted within 7 days from symptom onset, which is unique and different from the criterion of admission within 24 or 72 hours of symptom onset used in acute coronary syndrome registries of other countries.^21,22^ We observed the long prehospital delay and inefficient systems for first medical contact. A high proportion of patients with STEMI went to the hospital late, with only 72.0% presenting to the hospital within 12 hours from symptom onset, whereas more than 90% of such patients present to the hospital within 12 hours from symptom onset in the US.^23^ Moreover, ambulance transportation was used for only 19.4% of patients in provincial cities, 11.6% of patients in prefectures, and 12.0% of patients in counties, whereas 60% to 70% of patients in the US, Canada, the UK, and Japan and 50% of patients in Singapore use ambulances.^24,25,26,27,28,29^ The possible reasons for this are thought to be mainly patients’ being unaware of their AMI and lack of information on EMSs’ performance. Furthermore, EMSs are still underdeveloped and inconvenient in China, especially in rural and remote areas. Socioeconomic status was associated with a number of prehospital clinical, access-related, and transport variables that are associated with outcomes for patients with STEMI.^28^

Another important finding showed that the rate of early reperfusion therapy was markedly low (57.5% of all patients), which is lower than the rates of 71% in Sweden and 77% in the UK, but similar to the rates of 56% in Brazil and 53.9% in India.^1,16,21^ Among eligible patients who arrived at the hospital within 12 hours of symptom onset, 88.6%, 80.1%, and 72.6% of patients received reperfusion therapy in province-level, prefecture-level, and county-level hospitals, respectively, whereas the corresponding rates in the US are 96%, 94%, and 83% in the top, median, and bottom performing hospitals, respectively.^22^ The reasons for not receiving reperfusion therapy among the eligible patients mainly included patient factors (eg, concern about the safety of reperfusion or affordability) and physician factors (eg, diagnostic dilemma and clinical experience). Moreover, primary PCI, which is regarded as a more effective strategy in reperfusion therapy for STEMI, was used for most patients receiving reperfusion in province-level hospitals, whereas it was used much less frequently in county-level hospitals.

The most important finding in our study revealed that significant variation in the in-hospital mortality rate was observed for patients with STEMI in China, with the highest rate of 10.2% in county-level hospitals and the lowest rate of 3.1% in province-level hospitals. The remarkable, nearly 3-fold difference in mortality rate persisted in the subsets of patients stratified by presentation delay and reperfusion therapy. The higher mortality rate in county-level hospitals was associated with patients being in more critical condition, lower use of reperfusion therapy, and lack of advanced hospital facilities, such as CCUs and catheterization laboratories. Moreover, the odds of in-hospital death remained higher even after adjustment for these factors, implying that other immeasurable factors, such as insufficient capabilities in clinical expertise, also likely contribute to the much higher mortality in lower level hospitals.

For addressing the current gaps in the care of patients with STEMI especially in low-level hospitals in China as in other developing countries, efforts should be focused on widespread access to timely reperfusion, which is a proven paradigm with the greatest potential to improve survival, especially in rural areas that have a very low density of hospitals and limited resources.^30,31,32,33^ Fibrinolytic therapy is still encouraged in low-level hospitals or remote areas where primary PCI is less feasible. The establishment and staffing of CCUs and catheterization laboratories to provide the basic facilities are essential for addressing the prominent gaps.^34^ Moreover, officers and administrators from both government and hospital aspects should make more efforts to optimize the processes of reperfusion therapy for reducing in-hospital time delay.^35^ Intensive training and technical support for physicians and interventional cardiologists in the low-level hospitals should be implemented as well. In addition, more efforts should be made to educate residents about awareness of and prompt response to ischemic symptoms, and convenient and efficient EMS transportation for shortening prehospital delay should be made available. The EMSs should be strengthened to develop regional medical combination networks, which can improve patient prognosis independently of health care setting or geographical locations. The EMS-based strategy of transporting patients to existing PCI-capable hospitals is less costly and more effective than hospital expansion options.^23,25,36,37^ These insights provide implications for China as well as other developing countries as a world opportunity to narrow gaps and variations in the care and outcomes of patients with AMI. We can reference the evidence-based approaches such as the Acute Coronary Treatment and Intervention Outcomes Network^38^ and the Accelerator Project^39^ and develop useful and more effective models suitable for China for further practice and quality improvement.

### Limitations

This study has limitations that should be addressed. The CAMI registry did not include all hospitals and could not capture all the patients with STEMI. However, we uniquely included the 3 levels of hospitals instead of binary urban-rural or tertiary-secondary hospitals, which is in accordance with Chinese administrative and governmental models and objectively reflects routine practice and performance of AMI care in the Chinese medical system. The possibility of unmeasured confounding factors that can be present in any observational study exists. However, we tried to limit the effect of this by hierarchical multivariable models with adjustment for confounding factors. The study is not able to determine all of the factors associated with in-hospital mortality. However, our models are as comprehensive as the registry allowed and included the variables in multiple facets of presentation and care, consistent with or more extensive than other established registries. The CAMI registry enrolled patients admitted to the hospital but did not include outpatients. We analyzed the in-hospital mortality of patients with STEMI but could not assess out-of-hospital deaths.

## Conclusions

This cross-sectional study shows that there are significant variations in STEMI presentation, treatment patterns, and in-hospital outcomes among the 3 levels of hospitals in China. Multipronged and systematic efforts for quality improvement with the aim of delivering equitable management across all national hospitals from time of admission through to discharge and beyond are essential in a developing country with large population, limited resources, and large variation.

## Notes

Supplement. **eAppendix 1. **Full List of Hospitals in the China AMI Registry **eAppendix 2. **Definitions of In-Hospital Clinical Events **eTable 1. **Baseline Characteristics With 95% Confidence Intervals of Patients With STEMI Among the Three-Level Hospitals **eTable 2. **Multicomparisons in Baseline Characteristics of Patients With STEMI Among the Three-Level Hospitals in China **eTable 3. **Reperfusion Therapy and Medications During Hospitalization With 95% Confidence Intervals Among the Three-Level Hospitals **eTable 4. **Multicomparisons in Reperfusion Therapy and Medications During Hospitalization Among the Three-Level Hospitals in China **eTable 5. **In-Hospital Mortality of STEMI Patients Stratified by Subsets in China and Among the Three-Level Hospitals **eTable 6. **Multicomparison in In-Hospital Mortality Among the Three-Level Hospitals **eTable 7. **Adjusted In-Hospital Major Outcomes Risk Analysis in STEMI Patients Among the Three-Level Hospitals Based on Complete Data **eTable 8. **Adjusted In-Hospital Major Outcomes Risk Analysis in STEMI Patients Among the Three-Level Hospitals Based on Multiple Imputation Data **eTable 9. **Associated Factors With In-Hospital Mortality of STEMI Patients in China **eTable 10. **Associated Factors With In-Hospital Mortality of STEMI Patients in China Based on Multiple Imputation Data **eFigure 1. **Chinese Vertical Governmental and Administrative Model and the Three-Level Hospitals in the CAMI Registry **eFigure 2. **Reasons for No Reperfusion Therapy Among the Eligible STEMI Patients Admitted Within 12 Hours From Symptom Onset in China and Among the Three-Level Hospitals **eFigure 3. **Percentage of Patients With Door-to-Balloon Time ≤90 min and Door-To-Needle Time ≤30 min in Patients Who Received Primary Percutaneous Coronary Intervention and Fibrinolysis Respectively in China and Among Three-Level Hospitals
