# Supplementary material for: Association of Hospital-Level Differences in Care With Outcomes Among Patients With Acute ST-Segment Elevation Myocardial Infarction in China
Source: JAMA Netw Open. 2020 Oct 23;3(10):e2021677. doi: 10.1001/jamanetworkopen.2020.21677 (PMC7584928; doi:10.1001/jamanetworkopen.2020.21677)
Supplement: Supplement. — eAppendix 1. Full List of Hospitals in the China AMI Registry eAppendix 2. Definitions of In-Hospital Clinical Events eTable 1. Baseline Characteristics With 95% Confidence Intervals of Patients With STEMI Among the Three-Level Hospitals eTable 2. Multicomparisons in Baseline Characteristics of Patients With STEMI Among the Three-Level Hospitals in China eTable 3. Reperfusion Therapy and Medications During Hospitalization With 95% Confidence Intervals Among the Three-Level Hospitals eTable 4. Multicomparisons in Reperfusion Therapy and Medications During Hospitalization Among the Three-Level Hospitals in China eTable 5. In-Hospital Mortality of STEMI Patients Stratified by Subsets in China and Among the Three-Level Hospitals eTable 6. Multicomparison in In-Hospital Mortality Among the Three-Level Hospitals eTable 7. Adjusted In-Hospital Major Outcomes Risk Analysis in STEMI Patients Among the Three-Level Hospitals Based on Complete Data eTable 8. Adjusted In-Hospital Major Outcomes Risk Analysis in STEMI Patients Among the Three-Level Hospitals Based on Multiple Imputation Data eTable 9. Associated Factors With In-Hospital Mortality of STEMI Patients in China eTable 10. Associated Factors With In-Hospital Mortality of STEMI Patients in China Based on Multiple Imputation Data eFigure 1. Chinese Vertical Governmental and Administrative Model and the Three-Level Hospitals in the CAMI Registry eFigure 2. Reasons for No Reperfusion Therapy Among the Eligible STEMI Patients Admitted Within 12 Hours From Symptom Onset in China and Among the Three-Level Hospitals eFigure 3. Percentage of Patients With Door-to-Balloon Time ≤90 min and Door-To-Needle Time ≤30 min in Patients Who Received Primary Percutaneous Coronary Intervention and Fibrinolysis Respectively in China and Among Three-Level Hospitals [file jamanetwopen-e2021677-s001.pdf]

Supplemental Online Content

Xu H, Yang Y, Wang C, et al; China Acute Myocardial Infarction Registry Investigators. Association of hospital-level differences in care with outcomes among patients with acute ST-segment elevation myocardial infarction in China. *JAMA Netw Open*. 2020;3(10):e2021677. doi:10.1001/jamanetworkopen.2020.21677

eAppendix 1. Full List of Hospitals in the China AMI Registry

eAppendix 2. Definitions of In-Hospital Clinical Events

eTable 1. Baseline Characteristics With 95% Confidence Intervals of Patients With STEMI Among the Three-Level Hospitals

eTable 2. Multicomparisons in Baseline Characteristics of Patients With STEMI Among the Three-Level Hospitals in China

eTable 3. Reperfusion Therapy and Medications During Hospitalization With 95% Confidence Intervals Among the Three-Level Hospitals

eTable 4. Multicomparisons in Reperfusion Therapy and Medications During Hospitalization Among the Three-Level Hospitals in China

eTable 5. In-Hospital Mortality of STEMI Patients Stratified by Subsets in China and Among the Three-Level Hospitals

eTable 6. Multicomparison in In-Hospital Mortality Among the Three-Level Hospitals

eTable 7. Adjusted In-Hospital Major Outcomes Risk Analysis in STEMI Patients Among the Three-Level Hospitals Based on Complete Data

eTable 8. Adjusted In-Hospital Major Outcomes Risk Analysis in STEMI Patients Among the Three-Level Hospitals Based on Multiple Imputation Data

eTable 9. Associated Factors With In-Hospital Mortality of STEMI Patients in China

eTable 10. Associated Factors With In-Hospital Mortality of STEMI Patients in China Based on Multiple Imputation Data

eFigure 1. Chinese Vertical Governmental and Administrative Model and the Three-Level Hospitals in the CAMI Registry

© 2020 Xu H et al. *JAMA Network Open.*

eFigure 2. Reasons for No Reperfusion Therapy Among the Eligible STEMI Patients Admitted Within 12 Hours From Symptom Onset in China and Among the Three-Level Hospitals

eFigure 3. Percentage of Patients With Door-to-Balloon Time ≤90 min and Door-To-Needle Time ≤30 min in Patients Who Received Primary Percutaneous Coronary Intervention and Fibrinolysis Respectively in China and Among Three-Level Hospitals

This supplemental material has been provided by the authors to give readers additional information about their work.

© 2020 Xu H et al. *JAMA Network Open.*

eAppendix 1. Full List of Hospitals in the China AMI Registry

Hospital	Province/Municipality	City	PI
Fuwai Hospital	Beijing	Beijing	Yuan Wu
Beijing Friendship Hospital	Beijing	Beijing	Hongwei Li
Beijing Tongren Hospital	Beijing	Beijing	Changlin Lu
Beijing Daxing Hospital	Beijing	Daxing	Shujun Cao
Beijing Mentougou Hospital	Beijing	Mentougou	Dezhao Wang
Beijing Pinggu Hospital	Beijing	Pinggu	Guanglin Wei
Beijing Yanqing Hospital	Beijing	Yanqing	Jianbing Wang
Shanghai Jiaotong University Ruijin Hospital	Shanghai	Shanghai	Ruiyan Zhang
Shanghai 10th Hospital	Shanghai	Shanghai	Yawei Xu
Shanghai Fengxian Hospital	Shanghai	Fengxian	Zengyong Qiao
Tianjin Medical School General Hospital	Tianjin	Tianjin	Zheng Wan
Tianjin Baodi Hospital	Tianjin	Baodi	Yanjun Cao
Chongqing Medical School 2st Hospital	Chongqing	Chongqing	Yaohui Yin
Haerbin Medical School 1st Affiliated Hospital	Heilongjiang	Harbin	Weiming Li
Qiqihaer 1st Hospital	Heilongjiang	Qiqihar	Shuqing Wang
Tailai Hospital	Heilongjiang	Tailai	Gang Ma
Shuihua 1st Hospital	Heilongjiang	Shuihua	Yongchen Cai
Jilin University 1st Hospital	Jilin	Changchun	Yang Zheng
Tonghua Central Hospital	Jilin	Tonghua	Xuxia Zhang
Huinan County Hospital	Jilin	Huinan	Hongyan Guo
Shenyang Northern Hospital	Liaoning	Shenyang	Xiaozeng Wang
Fushun Central Hospital	Liaoning	Fushun	Ling Sun
Xiuyan County Hospital	Liaoning	Xiuyan	Jianhua Wu
Neimonggu Medical College 1st Affiliated Hospital	Inner Mongolia	Hohhot	Fengying Chen
Chifeng Hospital	Inner Mongolia	Chifeng	Ronghai Man
Aohan Hospital	Inner Mongolia	Aohan	Yanjie Li
Hebei Medcial School 2rd Affiliated Hospital	Hebei	Shijiazhuang	Xianghua Fu
Qinhuangdao 1st Hospital	Hebei	Qinhuangdao	Qingshen Wang
Qinhuangdao 2rd Hospital	Hebei	Changli	Liying Zhang
North-China Oil-administration General Hospital	Hebei	Renqiu	Xiaoli Gao
Changzhou Hospital	Hebei	Changzhou	Yali Hu
Hengshui Hardison Hospital	Hebei	Hengshui	Qun Zheng
Shanxi Cardiovascular Hospital	Shanxi	Taiyuan	Bao Li
Changzhi Hospital	Shanxi	Changzhi	Yuping zhang
Tunliu Hospital	Shanxi	Tunliu	Yaohong Dong
Henan Provincial Hospital	Henan	Zhengzhou	Chuanyu Gao
Linzhou Hospital	Henan	Linzhou	Zhoushun Qin
Changyuan Hospital	Henan	Changyuan	Guorui Hou
Xinxiang Central Hospital	Henan	Xinxiang	Lingling Liu
Yanjin Hospital	Henan	Yanjin	Shifeng Ren
Ye County hospital	Henan	Ye County	Dezhou wang
Pindingshan 2rd Hospital	Henan	Pindingshan	Xianting Luan
Anyang Prefecture Hospital	Henan	Anyang	Hui Liu
Puyang People’s Hospital	Henan	Puyang	Liping Ma

© 2020 Xu H et al. *JAMA Network Open.*

Hospital	Province/Municipality	City	PI
Xihua Hospital	Henan	Xihua	Chuntong Wang
Xi'an Jiaotong University 1st Hospital	Shan'xi	Xi'an	Zuyi Yuan
Weinan Central Hospital	Shan'xi	Weinan	Junnong Li
Jiuquan Hospital	Gansu	Jiuquan	Yaofeng Yuan
Jinta Hospital	Gansu	Jinta	Huide Liu
Ningxia Medical College General Hospital	Ningxia	Yinchuan	Shaobin jia
Wuzhong Hospital	Ningxia	Wuzhong	Xianghong Luo
Qinghai University Affiliated Hospital	Qinghai	Xining	Yin Liu
Qinhai Cardiovascular Hospital	Qinghai	Xining	Pinfa Liu
Xining 1st Hospital	Qinghai	Xining	Xianning Zhao
Hainan Prefectural Hospital of Qinghai	Qinghai	Gonghe	Bao Ma
Xinjiang Medical College 1st Affiliated Hospital	Xinjiang	Urumchi	Yitong Ma
Changji Hospital	Xinjiang	Changji	Mao Wang
Fukang Hospital	Xinjiang	Fukang	Shiming Gao
Urumchi Friendship Hospital	Xinjiang	Urumchi	Hang Lu
Shandong Provincial Hospital	Shandong	Jinan	Lianqun Cui
Taian Central Hospital	Shandong	Taian	Huanyi Zhang
Xintai Hospital	Shandong	Xintai	Hongyan Zhang
Nanjing University Gulou Hospital	Jiangsu	Nanjin	Biao Xu
Jiangsu North Hospital	Jiangsu	Yangzhou	Shenghu He
Xuzhou 1st Central Hospital	Jiangsu	Xuzhou	Qiang Fu
Jiangyan Hospital	Jiangsu	Jiangyan	Shihai Shen
Anhui Provincial Hospital	Anhui	Hefei	Likun Ma
Fuyang Hospital	Anhui	Fuyang	Bin Ning
Taihe Hospital	Anhui	Taihe	Jili Fan
Zhejiang University 2rd Affiliated Hospital	Zhejiang	Hangzhou	Yong Sun
Taizhou Enze medical Center	Zhejiang	Taizhou	Lijiang tang
Taizhou Hospital	Zhejiang	Linhai	Danlei Xu
Fujian Medical College Union Hospital	Fujian	Fuzhou	Lianglong Chen
Xiamen Heart Center	Fujian	Xiamen	Yan Wang
Fuqing Hospital	Fujian	Fuqing	Ping chen
Longyan 1st Hospital	Fujian	Longyan	Kaihong Chen
Wuhan Tongji Hospital	Hubei	Wuhan	Daowen wang
Jinzhou 1st Hospital	Hubei	Jinzhou	Shuixian peng
Tianmen 1st Hospital	Hubei	Tianmen	Shuping Wan
Gong'an Hospital	Hubei	Gongan	Laxi Zhang
Central South University Xiangya 2ndHospital	Hunan	Changsha	Shenhua Zhou
Xiangtan Central Hospital	Hunan	Xiangtan	Jianping Zeng
Xiangxiang Hospital	Hunan	Xiangxiang	Chonglun Zhou
Ya'an Hospital	Sichuan	Ya'an	Haibo zhang
Zigong 1st Hospital	Sichuan	Zigong	Dechao Zhong
Danleng County Hospital	Sichuan	Danleng	Yuquan Xiao
Guangxi Medical College 1st Affiliated Hospital	Guangxi	Nanning	Lang Li
Beihai Hospital	Guangxi	Beihai	Hai Zhu
Hepu Hospital	Guangxi	Hepu	Meisheng Lai
Nanchang Universuty 2ndAffiliated Hospital	Jiangxi	Nanchang	Xiaoshu Cheng
**Hospital**	**Province/Municipality**	**City**	**PI**

© 2020 Xu H et al. *JAMA Network Open.*

Pingxiang Hospital	Jiangxi	Pingxiang	Junming Ye
Shangli Hospital	Jiangxi	Shangli	Qishou Liu
Guizhou Cardiovascular Hospital	Guizhou	Guiyang	Tianhe Yang
Zhunyi 1st Hospital	Guizhou	Zhunyi	Zhengqiang Yuan
Honghuagang Hospital	Guizhou	Honghuagan g	Chengyuan Zhao
Pan County Hospital	Guizhou	Pan	Xianwen Jiang
Guangdong Provincial Hospital	Guangdong	Guangzhou	Jiyan Chen
Guangzhou Traditional Chinese Medical College 1st Affiliated Hospital	Guangdong	Guangzhou	Wei Wu
Jiangmen Hospital	Guangdong	Jiangmen	Gaoxing Zhang
Heshan Hospital	Guangdong	Heshan	Haiyuan Mai
Kunming Medical College 1st Affiliated Hospital	Yunnan	Kunming	Tao Guo
Yunnan St. John’s Hospital	Yunnan	Kunming	Yi Li
Chuxiong People’s Hosptal	Yunnan	Chuxiong	Xiaoming Liu
Yao’an Hospital	Yunnan	Yao’an	Jinlong Xu
Tibet People’s Hospital	Tibet	Lahsa	Gesang Luobu
Hainan Provincial Hospital	Hainan	Haikou	Bin Li
Sanya Hospital	Hainan	Sanya	Tiansong Wang
Wenchang Hospital	Hainan	Wenchang	Dong Wang

© 2020 Xu H et al. *JAMA Network Open.*

eAppendix 2. Definitions of In-Hospital Clinical Events

Name: Death

Patient died during this hospitalization. Date and primary cause should be noted.

Name: Primary cause of death:

1. Cardiovascular death indicates cause of death was sudden cardiac death, MI, unstable angina, or other CAD; vascular death (e.g., stroke, arterial embolism, pulmonary embolism, ruptured aortic aneurysm, or dissection); CHF; or cardiac arrhythmia
2. Non-cardiovascular death indicates cause of death was respiratory failure, pneumonia, cancer, trauma, suicide, or any other already defined cause (e.g., liver disease or renal failure)

Name: Heart Failure

Coding Instructions: Indicate if there is physician documentation or report of either new onset or acute reoccurrence of heart failure.

Supporting Definitions: Heart Failure:

Heart failure is defined as physician documentation or report of any of the following clinical symptoms of heart failure described as unusual dyspnea on light exertion, recurrent dyspnea occurring in the supine position, fluid retention; or the description of rales, jugular venous distension, pulmonary edema on physical exam, or pulmonary edema on chest x-ray presumed to be cardiac dysfunction. A low ejection fraction without clinical evidence of heart failure does not qualify as heart failure.

Name: Re-infarction

Indicate if there are clinical signs and symptoms of a new infarction or repeat infarction.

Target Value: Any occurrence between arrival at this facility and discharge

Supporting Definitions: Re-infarction:

Re-infarction occurs when there are clinical signs and symptoms of ischemia that is distinct from the presenting ischemic event. In patients where recurrent myocardial infarction is suspected from clinical signs or symptoms following the initial infarction, an immediate measurement of the employed cardiac marker is recommended. A second sample should be obtained 3–6 h later. Recurrent infarction is diagnosed if there is a20% increase of the value in the second sample. A 20% change should be considered significant, i.e. over that expected from analytical variability itself. This value should also exceed the 99th percentile URL. The ECG diagnosis of re-infarction following the initial infarction may be confounded by the initial evolutionary ECG changes. Re-infarction should be considered when ST elevation >0.1 mV reoccurs in a patient having a lesser degree of ST elevation or new pathological Q waves, in at least two contiguous leads, particularly when associated with ischemic symptoms for 20 min or longer.

Name: Arrhythmia

Indicate if the patient has a new episode or acute recurrence of arrhythmia in your facility documented by 1 of the following.

1. Arial fibrillation/flutter

2. Supraventricular tachycardia requiring treatment (supraventricular tachycardia that requires cardioversion, drug therapy, or is sustained for greater than 1 minute)

3. Ventricular tachycardia or ventricular fibrillation

4. Sinus pause or bradycardia

5. second-degree, or high-degree, or third-degree atrioventricular (AV) block

© 2020 Xu H et al. *JAMA Network Open.*

Name: Cardiac Arrest

Coding Instructions: Indicate if the patient experienced an episode of cardiac arrest in your facility.

Name: Cardiogenic Shock

Coding Instructions: Indicate if the patient had a new onset or acute recurrence of cardiogenic shock in your facility.

Note(s):

Transient episodes of hypotension reversed with IV fluid or atropine do not constitute cardiogenic shock. The hemodynamic compromise (with or without extraordinary supportive therapy) must persist for at least 30minutes.

Supporting Definitions: Cardiogenic shock:

Cardiogenic shock is defined as a sustained (>30 minutes) episode of systolic blood pressure <90 mm Hg, and/or cardiac index <2.2 L/min/m^2 determined to be secondary to cardiac dysfunction, and/or the requirement for parenteral inotropic or vasopressor agents or mechanical support (e.g., IABP, extracorporeal circulation, ventricular assist devices) to maintain blood pressure and cardiac index above those specified levels.

Name: Mechanical Complication

Rupture of the ventricular myocardium, as documented by cardiac echocardiography, ventriculography, pericardiocentesis, cardiac surgery, and/or autopsy. Rupture could be of the free wall or the ventricular septum. Included in this category is frank papillary muscle rupture.

Name: CVA/Stroke

Coding Instructions: Indicate if the patient experienced a stroke or cerebrovascular accident (CVA) in your facility.

Supporting Definitions: Stroke

A stroke or cerebrovascular accident is defined as loss of neurological function caused by an ischemic or hemorrhagic event with residual symptoms at least 24 hours after onset or leading to death.

Type of stroke:

1. Hemorrhagic: A stroke with documentation on imaging (e.g., CT scan or MRI of hemorrhage in the cerebral parenchyma, or a subdural or subarachnoid hemorrhage). Evidence of hemorrhagic stroke obtained from lumbar puncture, neurosurgery, or autopsy can also confirm the diagnosis.

2. Ischemic: A focal neurological deficit that results from a thrombus or embolus (and not due to hemorrhage) that appears and is still partially evident for more than 24 hours

3. Ischemic with hemorrhagic conversion

4. Unknown: if the type of stroke could not be determined by imaging or other means (from lumbar puncture, neurosurgery, or autopsy)

Name: Peripheral Arterial Event

Indicate if the patient experienced a peripheral arterial event in your facility.

Peripheral Arterial Event includes upper and lower extremity, renal, mesenteric, and abdominal aortic systems:

1. Acute limb ischemia, including acute ischemia caused by arterial emboli, arterial thrombosis, or arterial trauma from a vascular procedure with

a. Clinical history suggesting a rapid or sudden decrease in limb perfusion and

© 2020 Xu H et al. *JAMA Network Open.*

b. New pulse deficit with associated rest pain, pallor, parasthesias, or paralysis, or

c. Confirmation of arterial obstruction by imaging (including ultrasound, CT, MRI, or conventional angiography), surgical findings, or pathology

2. Acute arterial thrombosis or emboli of renal, or mesenteric, or retinal, or other peripheral arteries.

Name: Significant Bleeding Event

Indicate if there was a suspected or confirmed bleeding event observed and documented in the medical record that was associated with any of the following:

1. Hemoglobin drop of >=3 g/dL;

2. Transfusion of whole blood or packed red blood cells;

3. Procedural intervention/surgery at the bleeding site to reverse/stop or correct the bleeding (such as surgical closures/exploration of the arteriotomy site, balloon angioplasty to seal an arterial tear, endoscopy with cautery of a GI bleed).

4. Medical attention required to stop bleeding.

Name: Surgical Procedure or Intervention Required

Indicate if the suspected bleeding event observed required procedural intervention or surgery at the bleeding site to reverse, stop or correct the bleeding (e.g. surgical closures, exploration of the arteriotomy site, balloon angioplasty to seal an arterial tear, or endoscopy with cautery of a GI bleed).

Note(s):

Prolonged pressure does not qualify as an intervention, but ultrasonic guided compression after making a diagnosis of pseudoaneurysm does qualify.

Name: RBC/Whole Blood Transfusion

Indicate if there was a transfusion of either whole blood or packed red blood cells.

© 2020 Xu H et al. *JAMA Network Open.*

eTable 1. Baseline Characteristics With 95% Confidence Intervals of Patients With STEMI Among the Three-Level Hospitals

Characteristic	Province-level	Prefecture -level	County-level
>=75ys,%	15.0(13.9,16.1)	18.4(17.5,19.4)	23.6(21.8,25.6)
Male,%	79.5(78.3,80.8)	75.9(74.9,76.9)	66.3(64.2,68.4)
**Risk factorsand medical history**			
BMI>=25 kg/m^2,%	40.3(38.7,41.9)	33.7(32.6,34.9)	32.2(30.1,34.4)
Current smoker,%	51.1(49.5,52.7)	43.2(42.0,44.4)	39.9(37.7,42.1)
Hypertension,%	50.9(49.3,52.5)	48.7(47.5,49.9)	48.9(46.6,51.1)
Diabetes history,%	21.4(20.1,22.8)	18.6(17.7,19.6)	15.2(13.6,17.0)
Known dyslipidemia history,%	10.2(9.2,11.3)	5.9(5.3,6.5)	6.6(5.4,8.0)
Prior MI,%	6.9(6.1,7.8)	7.0(6.4,7.6)	6.0(5.0,7.2)
Prior HF, %	1.6(1.2,2.0)	1.5(1.2,1.8)	2.2(1.6,3.0)
Prior PCI, %	6.1(5.4,7.0)	4.4(3.9,4.9)	2.7(2.0,3.5)
Stroke, %	8.6(7.7,9.6)	9.3(8.6,10.0)	9.6(8.3,11.0)
PAD,%	0.6(0.4,1.0)	0.5(0.3,0.7)	0.1(0.0,0.4)
**Presentation**			
Means of transport,%			
Self/family	79.9(78.6,81.2)	87.2(86.4,88.0)	86.5(84.9,88.0)
Ambulance	19.4(18.1,20.7)	11.6(10.8,12.4)	12.0(10.6,13.5)
In-hospital	0.7(0.5,1.0)	1.3(1.0,1.6)	1.5(1.0,2.2)
Onset-to-arrival time			
Percentage,%			
<3h	28.9(27.5,30.4)	31.1(30.0,32.2)	33.2(31.2,35.4)
3-12h	46.3(44.8,47.9)	39.7(38.6,40.9)	36.5(34.4,38.7)
12-24h	8.1(7.3,9.0)	8.7(8.0,9.4)	9.7(8.4,11.0)
1-7 days	15.9(14.8,17.1)	19.6(18.7,20.6)	19.6(17.8,21.4)
Uncertain	0.7(0.5,1.0)	0.9(0.7,1.1)	1.0(0.6,1.6)
Anterior MI,%	52.0(50.4,53.5)	51.9(50.7,53.1)	53.8(51.5,56.0)
Heart failure on admission,%	12.2(11.2,13.3)	13.4(12.5,14.2)	17.9(16.2,19.7)
Cardiogenic shock on admission,%	2.8(2.3,3.3)	3.7(3.3,4.2)	6.6(5.5,7.8)
Cardiac arrest,%	1.3(1.0,1.7)	1.3(1.0,1.6)	2.1(1.5,2.9)
Killip class III/IV,%	6.0(5.3,6.8)	8.2(7.5,8.8)	9.5(8.2,10.8)

© 2020 Xu H et al. *JAMA Network Open.*

eTable 2. Multicomparisons in Baseline Characteristics of Patients With STEMI Among the Three-Level Hospitals in China

Characteristic	P value Provincial- vs Prefecture-level	P value Provincial- vs County-level	P value Prefecture vs County-level
Age, ys	<0.001	<0.001	<0.001
>=75ys,%	<0.001	<0.001	<0.001
Male,%	<0.001	<0.001	<0.001
**Risk factorsand medical history**			
BMI, kg/m^2	<0.001	<0.001	0.16
BMI>=25 kg/m^2,%	<0.001	<0.001	0.69
Current smoker,%	<0.001	<0.001	0.01
Diabetes history,%	0.002	<0.001	0.002
Known dyslipidemia history,%	<0.001	<0.001	1.00
Prior angina,%	<0.001	0.002	0.53
Prior PCI, %	<0.001	<0.001	0.001
Prior CABG, %	1.00	0.02	0.05
PAD,%	0.76	0.005	0.03
**Presentation**			
Means of transport,%<br>Self/family<br>Ambulance<br>In-hospital	<0.001	<0.001	0.12
Onset-to-arrival time<br>Percentage,%<br><3h<br>3-12h<br>12-24h<br>1-7 days<br>Uncertain	<0.001	<0.001	0.25
Heart failure on admission,%	0.30	<0.001	<0.001
Cardiogenic shock on admission,%	0.02	<0.001	<0.001
Cardiac arrest,%	1.00	0.05	0.02
Killip class III/IV,%	<0.001	<0.001	0.55

STEMI, ST-segment elevation myocardial infarction; BMI, body mass index; PCI, percutaneous coronary intervention; CABG, coronary artery bypass graft; PAD, peripheral artery disease.

© 2020 Xu H et al. *JAMA Network Open.*

eTable 3. Reperfusion Therapy and Medications During Hospitalization With 95% Confidence Intervals Among the Three-Level Hospitals

Characteristic	Province-level	Prefecture -level	County-level
**Reperfusion therapy**			
Among all the patients, n/N(%)	69.4(67.9,70.8)	54.3(53.1,55.5)	45.8(43.6,48.1)
Primary PCI	65.7(64.2,67.2)	42.2(41.0,43.4)	20.2(18.4,22.1)
Fibrinolysis	3.6(3.0,4.2)	11.9(11.1,12.7)	25.6(23.7,27.6)
Among the eligible patients admitted within 12 hours from onset, n/N(%)	88.6(87.3,89.8)	80.1(78.8,81.3)	72.6(70.0,75.2)
Primary PCI	83.8(82.4,85.2)	61.5(60.0,63.0)	31.6(29.0,34.4)
Fibrinolysis	4.7(3.9,5.6)	18.3(17.2,19.5)	41.0(38.2,43.9)
**Procedure**			
Coronary angiography, n/N(%)	75.2(73.8,76.5)	60.8(59.6,61.9)	32.1(30.1,34.2)
Stent implantation in primary PCI, n/N(%)	87.1(85.7,88.4)	84.2(82.8,85.6)	88.2(84.6,91.3)
DES	85.3(83.7,86.8)	93.6(92.5,94.6)	97.9(95.8,99.2)
Elective PCI, n/N(%)	20.0(18.7,21.3)	27.4(26.3,28.5)	15.4(13.9,17.1)
CABG, n/N(%)	0.8(0.5,1.1)	0.3(0.2,0.5)	0.2(0.0,0.4)
IABP use, n/N(%)	4.3(3.7,5.0)	3.4(3.0,3.8)	0.5(0.2,0.9)
**Medication during hospitalizaion**			
Aspirin, n/N(%)	97.6(97.0,98.0)	97.2(96.8,97.6)	96.2(95.3,97.0)
P_2Y_{12}-receptor inhibitor, n/N(%)	98.2(97.8,98.6)	97.8(97.4,98.1)	93.4(92.2,94.5)
Statin, n/N(%)	98.2(97.7,98.6)	97.9(97.5,98.2)	94.4(93.3,95.4)
β-blocker, n/N(%)	70.8(69.3,72.3)	70.7(69.6,71.8)	67.4(65.3,69.5)
ACEI/ARB, n/N(%)	59.7(58.2,61.3)	58.2(57.0,59.4)	61.5(59.3,63.7)
Heparin/Fondaparinux, n/N(%)	88.1(87.0,89.1)	93.1(92.5,93.7)	91.9(90.6,93.1)
GP IIb/IIIa inhibitor, n/N(%)	47.5(45.9,49.1)	31.9(30.8,33.1)	20.1(18.4,22.0)
Traditional Chinese Medicine, n/N(%)	13.7(12.7,14.9)	17.5(16.6,18.4)	17.4(15.7,19.1)

© 2020 Xu H et al. *JAMA Network Open.*

eTable 4. Multicomparisons in Reperfusion Therapy and Medications During Hospitalization Among the Three-Level Hospitals in China

Characteristic	P value Provincial- vs Prefecture-level	P value Provincial- vs County-level	P value Prefecture vs County-level
**Reperfusion therapy**			
Among all the patients,%	<0.001	<0.001	<0.001
Among the eligible patients admitted within 12 hours from onset,%	<0.001	<0.001	<0.001
**Procedure**			
Coronary angiography,%	<0.001	<0.001	<0.001
Emergent PCI,% (primary/rescue),%	<0.001	<0.001	<0.001
Elective PCI,%	<0.001	<0.001	<0.001
CABG,%	0.002	0.002	0.69
IABP use,%	0.05	<0.001	<0.001
Door-to needle time, min	0.04	0.01	0.41
Door-to-balloon time, min	0.001	1.00	0.02
**Medication during hospitalizaion**			
Aspirin,%	0.99	0.01	0.06
ADP receptor inhibitor,%	0.29	<0.001	<0.001
Statin,%	0.65	<0.001	<0.001
β-blocker,%	1.00	0.02	0.02
ACEI/ARB,%	0.41	0.60	0.03
UFH/LWH/Fondaparinux,%	<0.001	<0.001	0.22
GP IIb/IIIa inhibitor,%	<0.001	<0.001	<0.001
Traditional Chinese Medicine,%	<0.001	0.001	1.00

PCI, percutaneous coronary intervention; DES, drug-elute stent; CABG, coronary artery bypass graft; IABP, intra-aortic balloon pump; ACEI, angiotensin-converting enzyme inhibitor; ARB, angiotensin receptor blocker; GP, glucoprotein.

© 2020 Xu H et al. *JAMA Network Open.*

eTable 5. In-Hospital Mortality of STEMI Patients Stratified by Subsets in China and Among the Three-Level Hospitals

STEMI, ST-segment elevation myocardial infarction.

	**Total (n=12659)**	**Province-level (n=3974)**	**Prefecture-level (n=6722)**	**County-level (n=1963)**	**p value**
Mortality, n/N(%)	682/12659 (5.4)	123/3974 (3.1)	358/6722 (5.3)	201/1963 (10.2)	<0.001
Reperfusion					
Yes	237/7107 (3.3)	54/2652(2.0)	129/3572(3.6)	54/883(6.1)	<0.001
No	434/5227(8.3)	65/1167(5.6)	223/3009(7.4)	146/1051(13.9)	<0.001
Onset-to-arrival time					
≤12h	462/9114(5.1)	86/2993(2.9)	253/4752(5.4)	123/1369(9.0)	<0.001
>12h	209/3430(6.1)	36/953(3.8)	97/1902(5.1)	76/575(13.2)	<0.001

© 2020 Xu H et al. *JAMA Network Open.*

eTable 6. Multicomparison in In-Hospital Mortality Among the Three-Level Hospitals

Characteristic	P value Provincial- vs Prefecture-level	P value Provincial- vs County-level	P value Prefecture vs County-level
Overall	<0.001	<0.001	<0.001
Reperfusion			
Yes	<0.001	<0.001	0.004
No	0.09	<0.001	<0.001
Onset-to-arrival time			
<12h	<0.001	<0.001	<0.001
>12h	0.33	<0.001	<0.001

© 2020 Xu H et al. *JAMA Network Open.*

eTable 7. Adjusted In-Hospital Major Outcomes Risk Analysis in STEMI Patients Among the Three-Level Hospitals Based on Complete Data

Outcomes	Hospital level	Model	Adjusted OR (95%CI)	P value
Death	Province-level vs. prefecture-level	Unadjusted	1.76 (1.43-2.17)	<0.001
		Model 1:baseline characteristics	1.48 (1.17-1.88)	0.001
		Model 2:plus medical contact	1.53 (1.20-1.94)	<0.001
		Model 3:plus status at presentation	1.47 (1.14-1.89)	0.003
		Model 4:plus hospital facility	1.41(1.10-1.82)	0.007
		Model 5:plus reperfusion therapy	1.29 (1.00-1.67)	0.05
		Model 6: plus medication and IABP during hospitalization	1.39 (1.06-1.84)	0.02
	Province-level vs. county-level	Unadjusted	3.57 (2.83-4.50)	<0.001
		Model 1:baseline characteristics	2.75 (2.10-3.60)	<0.001
		Model 2:plus medical contact	2.83 (2.15-3.71)	<0.001
		Model 3:plus status at presentation	2.48 (1.85-3.31)	<0.001
		Model 4:plus hospital facility	1.60 (1.13-2.26)	0.01
		Model 5:plus reperfusion therapy	1.43 (1.00-2.03)	0.048
		Model 6: plus medication and IABP during hospitalization	1.43 (0.97-2.11)	0.07
	Prefecture-level vs. county-level	Unadjusted	2.03 (1.69-2.43)	<0.001
		Model 1:baseline characteristics	1.86 (1.51-2.29)	<0.001
		Model 2:plus medical contact	1.85 (1.50-2.28)	<0.001
		Model 3:plus status at presentation	1.69 (1.35-2.12)	<0.001
		Model 4:plus hospital facility	1.13 (0.84-1.52)	0.42
		Model 5:plus reperfusion therapy	1.11 (0.82-1.49)	0.51
		Model 6: plus medication and IABP during hospitalization	1.03 (0.74-1.42)	0.88
Heart Failure	Province-level vs. prefecture-level	Unadjusted	1.71 (1.51-1.94)	<0.001
		Model 1:baseline characteristics	1.41 (1.23-1.61)	<0.001
		Model 2:plus medical contact	1.42 (1.24-1.63)	<0.001
		Model 3:plus status at presentation	1.47 (1.26-1.71)	<0.001
		Model 4:plus hospital facility	1.49 (1.28-1.74)	<0.001
		Model 5:plus reperfusion therapy	1.48 (1.27-1.73)	<0.001
		Model 6: plus medication and IABP during hospitalization	1.57 (1.33-1.84)	<0.001
	Province-level vs. county-level	Unadjusted	2.85 (2.46-3.30)	<0.001
		Model 1:baseline characteristics	2.21 (1.87-2.61)	<0.001
		Model 2:plus medical contact	2.23 (1.89-2.64)	<0.001
		Model 3:plus status at presentation	2.19 (1.82-2.64)	<0.001
		Model 4:plus hospital facility	1.90 (1.54-2.36)	<0.001
		Model 5:plus reperfusion therapy	1.84 (1.48-2.29)	<0.001
		Model 6: plus medication and IABP during hospitalization	1.95 (1.55-2.45)	<0.001
	Prefecture-level vs. county-level	Unadjusted	1.66 (1.47-1.88)	<0.001
		Model 1:baseline characteristics	1.57 (1.37-1.80)	<0.001

© 2020 Xu H et al. *JAMA Network Open.*

Outcomes	Hospital level	Model	Adjusted OR (95%CI)	P value
		Model 2:plus medical contact	1.57 (1.36-1.80)	<0.001
		Model 3:plus status at presentation	1.49 (1.28-1.74)	<0.001
		Model 4:plus hospital facility	1.28 (1.06-1.54)	0.01
		Model 5:plus reperfusion therapy	1.24 (1.03-1.50)	0.02
		Model 6: plus medication and IABP during hospitalization	1.24 (1.02-1.51)	0.03
Cardiogenic shock	Province-level vs. prefecture-level	Unadjusted	1.69 (1.39-2.07)	<0.001
		Model 1:baseline characteristics	1.39 (1.11-1.73)	0.004
		Model 2:plus medical contact	1.45 (1.16-1.81)	0.001
		Model 3:plus status at presentation	1.41 (1.09-1.83)	0.009
		Model 4:plus hospital facility	1.39 (1.07-1.81)	0.01
		Model 5:plus reperfusion therapy	1.33 (1.02-1.73)	0.03
		Model 6: plus medication and IABP during hospitalization	1.54 (1.15-2.05)	0.003
	Province-level vs. county-level	Unadjusted	3.77 (3.03-4.70)	<0.001
		Model 1:baseline characteristics	2.81 (2.19-3.60)	<0.001
		Model 2:plus medical contact	2.95 (2.30-3.79)	<0.001
		Model 3:plus status at presentation	2.73 (2.03-3.68)	<0.001
		Model 4:plus hospital facility	1.79 (1.26-2.54)	0.001
		Model 5:plus reperfusion therapy	1.68 (1.18-2.41)	0.004
		Model 6: plus medication and IABP during hospitalization	1.95 (1.32-2.89)	<0.001
	Prefecture-level vs. county-level	Unadjusted	2.23 (1.88-2.65)	<0.001
		Model 1:baseline characteristics	2.02 (1.66-2.46)	<0.001
		Model 2:plus medical contact	2.04 (1.68-2.48)	<0.001
		Model 3:plus status at presentation	1.94 (1.53-2.45)	<0.001
		Model 4:plus hospital facility	1.29 (0.95-1.74)	0.11
		Model 5:plus reperfusion therapy	1.26 (0.93-1.72)	0.14
		Model 6: plus medication and IABP during hospitalization	1.27 (0.91-1.77)	0.16

Model 1: adjusting for patient characteristics (age, sex, hypertension, diabetes, prior myocardial infarction, prior heart failure).
Model 2: adjusting for model 1 plus medical contact (onset -to-arrival time, means of transport).
Model 3: adjusting for model 2 plus clinical status on admission (anterior-wall infarction, systolic blood pressure on admission, heart rate on admission, cardiogenic shock on admission, heart failure on admission, cardiac arrest prior to or on admission, Killip class).
Model 4: adjusting for model 3 plus hospital facility (including coronary care unit and coronary catheter lab availability).
Model 5: adjusting for model 4 plus reperfusion therapy.
Model 6: adjusting for model 5 plus medication use (aspirin, P_2Y_{12}-receptor inhibitor, statin, β-receptor inhibitor, angiotensin-converting enzyme inhibitor / angiotensin receptor blocker, intra-aortic balloon pump use during hospitalization).
STEMI, ST-segment elevation myocardial infarction; IABP, intra-aortic balloon pump.

© 2020 Xu H et al. *JAMA Network Open.*

eTable 8. Adjusted In-Hospital Major Outcomes Risk Analysis in STEMI Patients Among the Three-Level Hospitals Based on Multiple Imputation Data

Outcomes	Hospital level	Model	Adjusted OR (95%CI)	P value
Death	Province-level vs. prefecture-level	Model 1:baseline characteristics	1.64 (1.33-2.03)	<0.001
		Model 2:plus medical contact	1.69 (1.36-2.09)	<0.001
		Model 3:plus status at presentation	1.61 (1.29-2.01)	<0.001
		Model 4:plus hospital facility	1.57(1.26-1.96)	<0.001
		Model 5:plus reperfusion therapy	1.44 (1.15-1.80)	0.001
		Model 6: plus medication and IABP during hospitalization	1.55 (1.23-1.95)	<0.001
	Province-level vs. county-level	Model 1:baseline characteristics	3.01 (2.37-3.81)	<0.001
		Model 2:plus medical contact	3.08 (2.43-3.91)	<0.001
		Model 3:plus status at presentation	2.63 (2.05-3.39)	<0.001
		Model 4:plus hospital facility	1.87 (1.38-2.53)	<0.001
		Model 5:plus reperfusion therapy	1.68 (1.24-2.28)	<0.001
		Model 6: plus medication and IABP during hospitalization	1.87 (1.36-2.56)	<0.001
	Prefecture-level vs. county-level	Model 1:baseline characteristics	1.83 (1.52-2.21)	<0.001
		Model 2:plus medical contact	1.83 (1.52-2.20)	<0.001
		Model 3:plus status at presentation	1.64 (1.34-2.00)	<0.001
		Model 4:plus hospital facility	1.19 (0.92-1.54)	0.41
		Model 5:plus reperfusion therapy	1.16 (0.90-1.51)	0.25
		Model 6: plus medication and IABP during hospitalization	1.21 (0.92-1.58)	0.17
Heart Failure	Province-level vs. prefecture-level	Model 1:baseline characteristics	1.63 (1.44-1.85)	<0.001
		Model 2:plus medical contact	1.63 (1.44-1.86)	<0.001
		Model 3:plus status at presentation	1.67 (1.46-1.91)	<0.001
		Model 4:plus hospital facility	1.71 (1.49-1.96)	<0.001
		Model 5:plus reperfusion therapy	1.67 (1.46-1.92)	<0.001
		Model 6: plus medication and IABP during hospitalization	1.74 (1.51-2.00)	<0.001
	Province-level vs. county-level	Model 1:baseline characteristics	2.50 (2.15-2.91)	<0.001
		Model 2:plus medical contact	2.49 (2.14-2.91)	<0.001
		Model 3:plus status at presentation	2.42 (2.05-2.87)	<0.001
		Model 4:plus hospital facility	2.02 (1.66-2.45)	<0.001
		Model 5:plus reperfusion therapy	1.97 (1.62-2.40)	<0.001
		Model 6: plus medication and IABP during hospitalization	2.11 (1.73-2.58)	<0.001
	Prefecture-level vs. county-level	Model 1:baseline characteristics	1.53 (1.35-1.74)	<0.001
		Model 2:plus medical contact	1.53 (1.34-1.73)	<0.001
		Model 3:plus status at presentation	1.45 (1.26-1.67)	<0.001
		Model 4:plus hospital facility	1.18 (1.00-1.41)	0.06
		Model 5:plus reperfusion therapy	1.18 (0.99-1.40)	0.06

© 2020 Xu H et al. *JAMA Network Open.*

		Model 6: plus medication and IABP during hospitalization	1.21 (1.02-1.44)	0.03
Outcomes	Hospital level	Model	Adjusted OR (95%CI)	P value
Cardiogenic shock	Province-level vs. prefecture-level	Model 1:baseline characteristics	1.59 (1.30-1.95)	<0.001
		Model 2:plus medical contact	1.66 (1.36-2.04)	<0.001
		Model 3:plus status at presentation	1.55 (1.23-1.95)	<0.001
		Model 4:plus hospital facility	1.54 (1.23-1.94)	<0.001
		Model 5:plus reperfusion therapy	1.50 (1.19-1.89)	<0.001
		Model 6: plus medication and IABP during hospitalization	1.68 (1.32-2.14)	<0.001
	Province-level vs. county-level	Model 1:baseline characteristics	3.28 (2.62-4.10)	<0.001
		Model 2:plus medical contact	3.43 (2.74-4.29)	<0.001
		Model 3:plus status at presentation	3.02 (2.33-3.92)	<0.001
		Model 4:plus hospital facility	2.02 (1.48-2.75)	<0.001
		Model 5:plus reperfusion therapy	1.94 (1.42-2.66)	<0.001
		Model 6: plus medication and IABP during hospitalization	2.32 (1.67-3.21)	<0.001
	Prefecture-level vs. county-level	Model 1:baseline characteristics	2.06 (1.73-2.45)	<0.001
		Model 2:plus medical contact	2.06 (1.73-2.45)	<0.001
		Model 3:plus status at presentation	1.95 (1.58-2.40)	<0.001
		Model 4:plus hospital facility	1.31 (1.00-1.71)	0.05
		Model 5:plus reperfusion therapy	1.30(0.99-1.70)	0.06
		Model 6: plus medication and IABP during hospitalization	1.38 (1.04-1.82)	0.02

Model 1: adjusting for patient characteristics (age, sex, hypertension, diabetes, prior myocardial infarction, prior heart failure).
Model 2: adjusting for model 1 plus medical contact (onset -to-arrival time, means of transport).
Model 3: adjusting for model 2 plus clinical status on admission (anterior-wall infarction, systolic blood pressure on admission, heart rate on admission, cardiogenic shock on admission, heart failure on admission, cardiac arrest prior to or on admission, Killip class).
Model 4: adjusting for model 3 plus hospital facility (including coronary care unit and coronary catheter lab availability).
Model 5: adjusting for model 4 plus reperfusion therapy.
Model 6: adjusting for model 5 plus medication use (aspirin, P_2Y_{12}-receptor inhibitor, statin, β-receptor inhibitor, angiotensin-converting enzyme inhibitor / angiotensin receptor blocker, intra-aortic balloon pump use during hospitalization).

© 2020 Xu H et al. *JAMA Network Open.*

eTable 9. Associated Factors With In-Hospital Mortality of STEMI Patients in China

Factors	Adjusted OR (95%CI)	P value
**Hospital level**		
Prefecture-level (vs Province-level hospital)	1.39 (1.06-1.84)	0.02
County-level (vs Province-level hospital)	1.43(0.97-2.11)	0.07
**Hospital facility**		
CCU	0.62 (0.41-0.92)	0.02
Coronary catheter lab availability	0.47 (0.31-0.72)	<0.001
**Demography**		
Male gender	0.76 (0.61-0.95)	0.01
Age (10years)	1.46 (1.34-1.60)	<0.001
**Risk factor**		
Hypertension	1.51 (1.22-1.87)	<0.001
Diabetes	1.50 (1.17-1.91)	0.001
**Medical contact**		
Onset -to-arrival time >12 hours	0.71 (0.55-0.91)	0.01
**Presentation**		
Anterior-wall infarction	1.64(1.31-2.05)	<0.001
SBP on admission	0.88(0.84-0.92)	<0.001
HR on admission	1.01 (1.01, 1.02)	<0.001
Cardiogenic shock on admission	1.72 (1.09-2.71)	0.02
Heart failure on admission	1.41 (1.03-1.93)	0.03
Cardiac arrest prior to or on admission	2.37 (1.27-4.39)	0.01
Killip class III	1.62 (1.06-2.48)	0.03
Killip class IV	1.69 (1.06-2.70)	0.03
**Treatment**		
Reperfusion therapy	0.57 (0.45-0.74)	<0.001
Statin	0.29 (0.19-0.44)	<0.001
β-blocker	0.55 (0.44-0.69)	<0.001
ACEI/ARB	0.54 (0.43-0.68)	<0.001

Adjustment variates: age, sex, hypertension, diabetes, prior myocardial infarction, prior heart failure, onset -to-arrival time, means of transport, anterior-wall infarction, SBP on admission, HR on admission, cardiogenic shock on admission, heart failure on admission, cardiac arrest prior to or on admission, Killip class, CCU, coronary catheter lab availability, reperfusion therapy, aspirin, P_2Y_{12}- receptor inhibitor, statin, β-blocker, ACEI/ARB, intra-aortic balloon pump use during hospitalization. STEMI, ST-segment elevation myocardial infarction; SBP, systolic blood pressure; HR, heart rate; CCU, coronary care unit; ACEI, angiotensin-converting enzyme inhibitor; ARB, angiotensin receptor blocker.

© 2020 Xu H et al. *JAMA Network Open.*

eTable 10. Associated Factors With In-Hospital Mortality of STEMI Patients in China Based on Multiple Imputation Data

Factors	Adjusted OR (95%CI)	P value
**Hospital level**		
Prefecture-level (vs Province-level hospital)	1.55 (1.23-1.95)	<0.001
County-level (vs Province-level hospital)	1.87 (1.36-2.56)	<0.001
**Hospital facility**		
CCU	0.70 (0.49-1.00)	0.049
Coronary catheter lab availability	0.60 (0.43-0.85)	0.004
**Demography**		
Male gender	0.80 (0.66-0.96)	0.02
Age (10years)	1.04 (1.03-1.05)	<0.001
**Risk factor**		
Hypertension	1.44 (1.20-1.73)	<0.001
Diabetes	1.47 (1.18-1.83)	<0.001
**Medical contact**		
Onset -to-arrival time >12 hours	0.68 (0.55-0.85)	<0.001
**Presentation**		
Anterior-wall infarction	1.53 (1.27-1.86)	<0.001
SBP on admission	0.99 (0.98-0.99)	<0.001
HR on admission	1.02 (1.01, 1.02)	<0.001
Cardiogenic shock on admission	1.81 (1.25-2.60)	0.002
Heart failure on admission	1.27(0.97-1.65)	0.08
Cardiac arrest prior to or on admission	2.85 (1.80-4.51)	<0.001
Killip class III	1.48 (1.02-2.16)	0.04
Killip class IV	1.72 (1.18-2.51)	0.005
**Treatment**		
Reperfusion therapy	0.58 (0.47-0.72)	<0.001
Statins	0.28 (0.19-0.39)	<0.001
β-blocker	0.57 (0.47-0.69)	<0.001
ACEI/ARB	0.52 (0.42-0.63)	<0.001

Adjustment variates: age, sex, hypertension, diabetes, prior myocardial infarction, prior heart failure, onset -to-arrival time, means of transport, anterior-wall infarction, SBP on admission, HR on admission, cardiogenic shock on admission, heart failure on admission, cardiac arrest prior to or on admission, Killip class, CCU, coronary catheter lab availability, reperfusion therapy, aspirin, P_2Y_{12}- receptor inhibitor, statin, β-blocker, ACEI/ARB, intra-aortic balloon pump use during hospitalization. STEMI, ST-segment elevation myocardial infarction; SBP, systolic blood pressure; HR, heart rate; CCU, coronary care unit; ACEI, angiotensin-converting enzyme inhibitor; ARB, angiotensin receptor blocker.

© 2020 Xu H et al. *JAMA Network Open.*

eFigure 1. Chinese Vertical Governmental and Administrative Model and the Three-Level Hospitals in the CAMI Registry

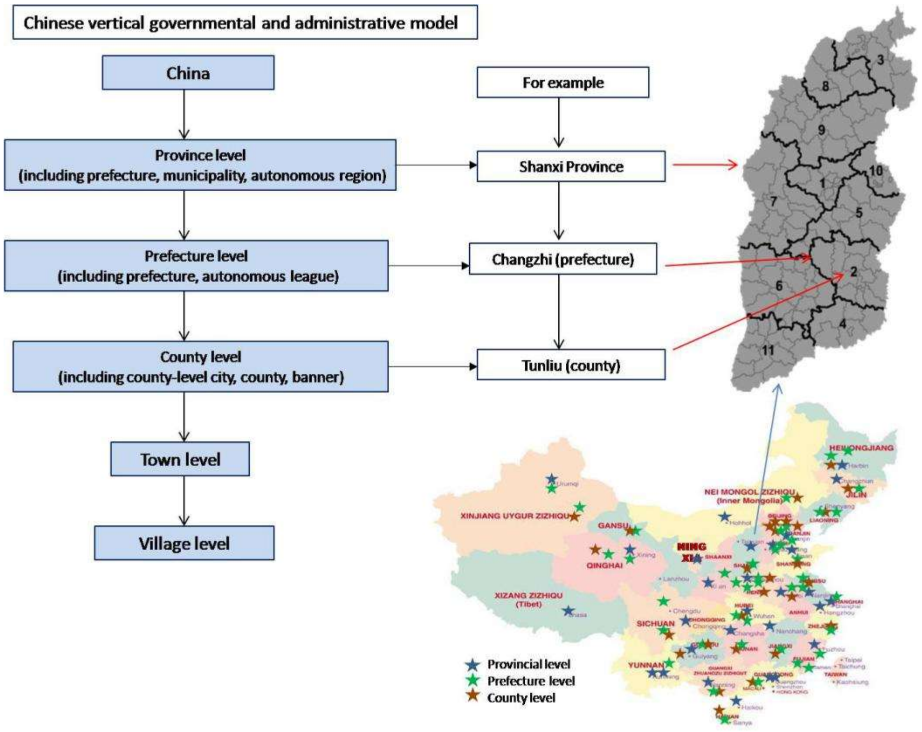

For example Shanxi Province. Red arrows point to the whole Shanxi province, the selected Changzhi Prefecture and Tunliu County, from top to botton respectively. The blue arrow points to the location of Shanxi Province in the hospital distribution map of CAMI registry.

© 2020 Xu H et al. *JAMA Network Open.*

eFigure 2. Reasons for No Reperfusion Therapy Among the Eligible STEMI Patients Admitted Within 12 Hours From Symptom Onset in China and Among the Three-Level Hospitals

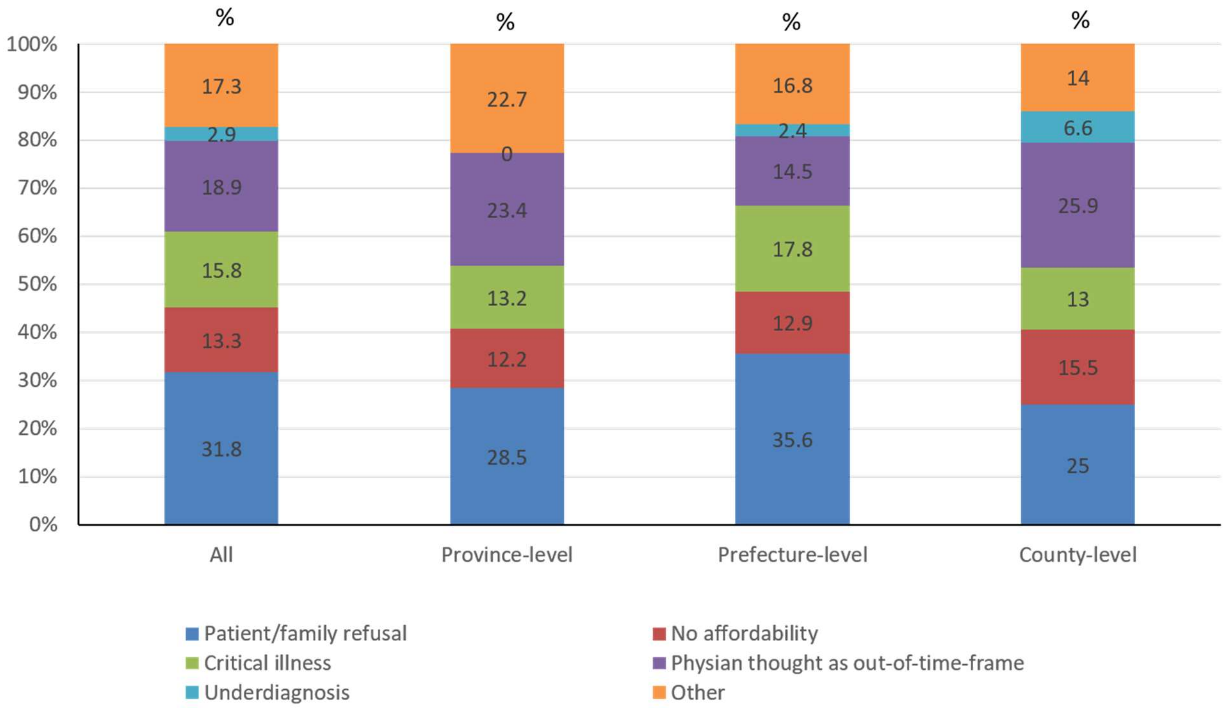

STEMI, ST-segment elevation myocardial infarction.

© 2020 Xu H et al. *JAMA Network Open.*

eFigure 3. Percentage of Patients With Door-to-Balloon Time ≤90 min and Door-To-Needle Time ≤30 min in Patients Who Received Primary Percutaneous Coronary Intervention and Fibrinolysis Respectively in China and Among Three-Level Hospitals

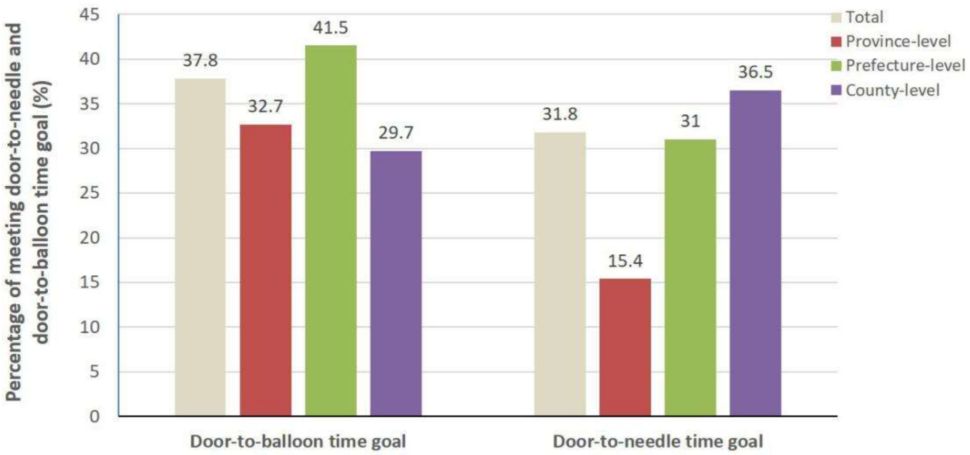

© 2020 Xu H et al. *JAMA Network Open.*
